# Barriers and facilitators in identifying and addressing perinatal mood disorders: a healthcare provider perspective

**DOI:** 10.3389/fgwh.2026.1880598

**Published:** 2026-07-15

**Authors:** Kayleigh Gregory, Aubrey Jones, Lindsay Thompson, Karli Adams, Madelyn Hill, Mastano N. Dzimbiri

**Affiliations:** 1 Family Science and Social Work, Miami University, Oxford, OH, United States; 2 College of Social Work, University of Kentucky, Lexington, KY, United States; 3 College of Health Sciences and Professions, Ohio University, Athens, OH, United States

**Keywords:** healthcare perspective, maternal health, PMAD, postpartum depression, referral, screening barriers

## Abstract

**Background:**

Perinatal mood and anxiety disorders (PMAD's) are the leading cause of maternal mortality, and one of the most common medical complications identified during the perinatal experience. Research indicates that the early identification of PMADs can result in more effective management of mental health conditions. By examining healthcare professionals' perceptions of the barriers and facilitators to these conversations, we can identify what helps or hinders effective dialogue. This understanding not only informs training and practice but also strengthens the use of screening tools, ensuring they are applied in ways that are meaningful, accessible, and responsive to patient needs.

**Methods:**

We used a purposive sampling technique to recruit 19 providers who work in the United States; specifically, Ohio, Kentucky, and Indiana. Semi-structured interviews with open-ended interview questions were conducted. Qualitative data were analyzed using thematic analysis, and main themes emerged from the transcripts.

**Results:**

Four major themes were identified regarding screening barriers and facilitators for mental health care observed in OBGYN practice in the Midwest area: (1) Approaches to Screening and Assessment, (2) Provider Responses and Interventions, (3) Facilitators of Engagement and, (4) Barriers at Multiple Levels.

**Conclusion:**

Study findings provide a comprehensive view of the screening barriers and facilitators perceived by these healthcare professionals during the perinatal period. These findings offer insight into how provider perspectives influence the implementation of mental health screening practices. Overall, results may offer evidence for improving screening protocols, provider training, and patient-centered approaches to perinatal mental health care.

## Introduction

Perinatal mood and anxiety disorders (PMADs) describe several mental health symptoms and conditions experienced by pregnant and postpartum individuals (1, 2). PMADs are the most common complication associated with childrearing and include anxiety, depression, bipolar, obsessive-compulsive disorder, psychosis, and post-traumatic stress disorder (3, 4). If left untreated, PMADs can have lifelong consequences for child development, family well-being, and economic advancement (1, 2, 5). Beyond their profound impact on individuals and families, PMADs also impose an economic burden on society, with an estimated cost of $14 billion over 5 years in the United States, driven primarily by lost productivity and healthcare expendures (6). Importantly, the severity of these conditions is underscored by the fact that mental health conditions, including suicide and overdose, have emerged as the leading cause of pregnancy-related deaths (2, 7–10).

Early identification can result in more effective management of PMADs (1, 4) allowing for quicker transitions into supportive interventions, reducing the risk of a potential escalation (1). One way of early identification is through patient screening. The American College of Obstetricians and Gynecologists (3) recommends screening for PMADs at multiple visits throughout the perinatal period. Other research supports the practice of screening in both perinatal and pediatric appointments (5). Obstetricians, nurses, pediatricians, midwives, and physician assistants are identified by various organizations and researchers as providers positioned to screen and identify individuals experiencing PMADs (5, 11, 12). These designated providers commonly utilize screening instruments to identify PMADs during these outlined medical visits. ACOG encourages the use of screening tools that are standardized and validated (3) including the Edinburgh Postnatal Depression Scale (EPDS) and the Patient Health Questionnaire-9 (PHQ-9).

### Barriers to screening

Despite validated screening instruments and trained medical personnel, there remains barriers to effective screening and referral of patients when it comes to PMADs. One barrier is the stigma associated with mental health. Garapati and colleagues (1)^(p8–9)^ quantify stigma as the “negative attitudes, beliefs, and stereotypes” society holds about individuals with mental health challenges such as PMADs. Research has shown that internalized negative perceptions about seeking support as well as fear of external judgment, professional repercussions, and removal of the child from the home, if diagnosed, serve as barriers to effective perinatal screening and referral (2, 11, 13). Perinatal individuals experiencing PMADs may not fully disclose mental health concerns due to these fears, hindering the effectiveness of screening devices.

Other barriers to screening and referral include reduced access to medical care. Limited access to transportation can prevent pregnant and postpartum individuals from attending critical medical appointments, which reduces the likelihood of the identification of PMADs and early implementation of interventions and supports (1, 13). Financial barriers can also reduce access to medical care. Poverty and insufficient insurance coverage can greatly reduce timely access to medical services and consequential opportunities to identify and address PMADs (1, 2, 13, 14).

Language and cultural barriers further challenge the identification and referral process of individuals experiencing PMADs. In addition to challenges in communication between patient and provider, screeners such as the EPDS developed in British English may provide difficulty with translation accuracy as well as to populations with lower reading levels (15). Cultural barriers include challenges in the provision of competent care, unrealized perception differences of PMADs, and the consequential disparities in treatment (1, 13).

Providers and healthcare institutions may encounter barriers to screening and referring patients experiencing PMADs due to shortages in time and resources. Screening practices may be omitted or implemented against best practice due to timing concerns, lack of administrative follow through, or a shortage of trained practitioners (2, 15). Providers may not have the resources to refer individuals to or are concerned screening will lead to a “Pandora's box” that will not be handled within the time and resource constraints (15).^(p2)^ These limitations in personnel, resources, and time can limit the use of screening tools.

Additionally, provider attitudes can serve as a barrier to the screening and referral process. If the provider is perceived to lack empathy or sensitivity in conversations, then a patient may refrain from sharing openly about mental health concerns (11, 16). Research has also noted that providers who are perceived to be dismissive of mental concerns or more adept with physical ailments may also contribute to this screening and referral barrier (13, 16).

A further barrier to the screening and referral process is limited provider knowledge. If practitioners are unaware of validated screening instruments or evidence-based support resources, medical institutions will likely struggle with the identification and treatment of PMADs (1). Similarly, even if practitioners are using validated and evidence-based practices, lack of knowledge or training may lead to the incorrect application of these tools. The EPDS, for example, employs a specific scoring method that may lead less knowledgeable administrators to incorrect conclusions in the screening process (15).

While various factors can act as barriers to the screening and referral process, research has also identified factors that help providers in these processes. Practice Guidelines such as those set out by the Association of Women's Health, Obstetric and Neonatal Nurses (AWHONN) or the American College of Obstetricians and Gynecologists (ACOG) help standardize best practices for the identification, diagnosis, and treatment of PMADs (3, 5). Within these Practice Guidelines, standardized screening instruments, evidence-based practices, and key medical visits are outlined and suggested for screening and referral practices (3, 5).

### Gaps for diverse populations

Gaps exist in the identification and treatment for individuals experiencing PMADs. For example, PMADs were found to be diagnosed more frequently in urban settings at 19.5% compared to 18.0% frequency in rural areas (17). When looking at ethnic and racially diverse populations, research has discovered further gaps in PMADs screening and treatment. Taiwo and colleagues (2024) have found that PMAD screening, when stratified by ethnic and racial criteria, occurred significantly less frequently for Latina mothers (12). Additionally, Indigenous mothers were found to be three times more likely than other groups to have unmet mental health needs and not receive counseling. Black mothers were found to be almost two times more likely than other groups to have unmet mental health needs and not receive counseling (12). However, Black and White mothers were found to more frequently seek mental health consultation than mothers from other ethnic and racial groups (18). Further findings noted that “Asian; Native Hawaiian or Pacific Islander; Southwest Asian, Middle Eastern, or North African; Hispanic; and non-Hispanic Black” mothers received mental health support significantly less frequently than non-Hispanic White mothers (19).^(p486)^ In this way, significant gaps have been identified between PMAD identification and treatment between various racial and ethnic groups. When citizenship is considered, research has highlighted the various challenges in securing healthcare and mental health support encountered by parents who are immigrants or refugees (12). In terms of gender and sexually diverse populations, research has shown individuals often encounter “multiple complex mental health challenges” during pregnancy and postpartum periods (14).^(p1635)^ On top of these challenges, studies have noted individuals within this community may not be adequately included or supported in mental health conversations due to provider gaps in knowledge.

### Research study

While prior studies have explored healthcare providers' experiences with perinatal mental health care, important gaps remain regarding how providers across diverse professional roles with variety of past training perceive barriers and facilitators to the management of PMADs. Addressing this gap may inform the development of strategies to improve screening, referral and treatment pathways. Therefore, this study aimed to explore the clinical perspective of the barriers and facilitators in identifying and addressing perinatal mood disorders.

## Methods

The study used semi-structured, focused qualitative interviews. A grounded theory approach was applied, with interview questions designed following an extensive review of literature examining providers' experiences in women's health care. All confidential interviews were conducted and recorded virtually using a secure, research compliant, Internet-based conferencing tool provided by the PIs institution. The interviews averaged 31 min in length with a range of 20–60 min per interview. For the purpose of this paper, the analysis focused specifically on responses outlined below. This approach provided a deeper exploration of key themes related to screening barriers and facilitators. This study used the Standards for Reporting Qualitative Research (SRQR) items to follow reporting guidelines for qualitative research. The questions were followed by probes to elicit further discussion. Ethical approval for this study was obtained from XXXXX [institutional review board (IRB) number: 02230r].

## Participant recruitment

Providers were recruited in early 2024. Participants were eligible if they were over 18 years old, able to complete an interview virtually, work as an obstetrician-gynecologist (OB-GYN), Midwife, Medical Doctor (MD) or Nurse Practitioner (NP) with the perinatal population in either Ohio, Kentucky or Indiana. Recruitment initially employed purposive sampling. As enrollment progressed, snowball sampling was utilized to identify additional eligible participants through professional networks. Recruitment challenges were encountered due to time constraints among providers, and limited responses, which reduced participation availability. Snowball sampling was implemented to increase participant enrollment. The PI and graduate assistants (MD, MH) contacted providers by phone and email across more than 50 local hospitals and clinics to personally invite women's health providers to participate. Additional recruitment strategies included the use of Research Match and social media pages. Interested providers were instructed to email the PI for further information. Eligible respondents received a research information sheet detailing the study and potential scheduling options for a virtual interview. Providers who consented to participate completed individual interviews, which continued until thematic saturation was reached (20).

## Procedure

All interviews were conducted virtually via Zoom, a secure platform provided by the PI's institution, and were audio-recorded with participant consent. Prior to each interview, the interviewer reviewed the oral consent form, which had also been emailed to participants in advance. All providers gave verbal consent to participate and to be recorded before beginning the session. As compensation, participants received a $25 Amazon e-gift card. Between January 3 and April 25, 2024, the PI conducted 15 interviews independently and an additional 4 with a trained study team member. Following completion, the PI generated preliminary transcripts from the recordings (approximately 70%–80% accurate), which were subsequently reviewed and corrected by study team members to ensure accuracy.

## Data analysis

Directed content analysis was conducted following Braun and Clarke's (2006) six-step iterative framework for thematic analysis (21). The PI and two trained graduate students, who co-conducted the interviews, began with five randomly selected transcripts. Each researcher independently reviewed the transcripts, familiarized themselves with the data, conducted open coding, and generated preliminary codes. The team then met to compare coding, resolve discrepancies, and reach consensus on the most appropriate codes to capture the data. An audit trail was maintained to define codes and document coding decisions. The remaining 14 transcripts were randomly assigned to the three researchers for independent coding using the agreed-upon guidelines, then themes were developed to reflect key aspects of providers' experiences. To strengthen rigor, all transcripts were reviewed a second time. Additionally, the team compiled a glossary of terms and shared it with providers for review and feedback, though only limited responses were received beyond expressions of gratitude for the project (22).

## Results

The characteristics of the study population are shown in Table 1. Data saturation was reached and verified after a total of 19 interviews. Providers (*N* = 19) were Doctors of Medicine (*n* = 9), Certified Nurse Midwives (*n* = 5), Doctors of Osteopathic Medicine (*n* = 3), Nurse Practitioner (*n* = 1) and Midwives (*n* = 1). Providers were of the following racial backgrounds: White/Caucasian (*n* = 17), Asian/Pacific Islander (*n* = 1), and Black/African American (*n* = 1). Providers self-identified as primarily female (*n* = 17) followed by male (*n* = 2). Providers' age range was 29–53 years and time spent in their current role ranged from 1 to 24 years. All providers stated they were satisfied or very satisfied in their current position.

**Table 1:** Characteristics of the study population (*N* = 19).

Age	Gender	Race	Current Job Title	Years of Experience	Years in Current Role	Satisfaction in Current Position
44	Female	White/Caucasian	OB GYN/MD	15	5	Very Satisfied
30	Female	Asian/Pacific Islander	OB GYN/MD	5	1	Satisfied
34	Male	White/Caucasian	OB GYN/MD	6	6	Very Satisfied
31	Female	White/Caucasian	OB GYN/MD	5.5	5.5	Satisfied
42	Female	White/Caucasian	OB GYN/MD	17	13	Very Satisfied
30	Female	White/Caucasian	OB GYN/MD Resident	4	4	Satisfied
53	Male	White/Caucasian	OB GYN/MD	13	13	Satisfied
47	Female	White/Caucasian	OB GYN/MD Resident	24	24	Satisfied
29	Female	White/Caucasian	OB GYN/MD Resident	4	4	Very Satisfied
46	Female	White/Caucasian	CNM	14	14	Satisfied
44	Female	Black/African American	CNM	7	15	Very Satisfied
50	Female	White/Caucasian	CNM	25	20	Satisfied
53	Female	White/Caucasian	CNM	25	20	Very Satisfied
42	Female	White/Caucasian	CNM	2	19	Satisfied
40	Female	White/Caucasian	OB GYN/DO	13	13	Satisfied
41	Female	White/Caucasian	OB GYN/DO	13	13	Satisfied
31	Female	White/Caucasian	OB GYN/DO	5	5	Satisfied
53	Female	White/Caucasian	NP	20	2	Satisfied
42	Female	White/Caucasian	Midwife	20	16	Satisfied

OB GYN, obstetrician and gynecologist; DO, doctor of osteopathic medicine; MD, doctor of medicine; NP, nurse practitioner; CNM, certified nurse midwife; WHNP, women's health nurse practitioner.

## Overview of results

Thematic analysis of the interviews focused on the following questions for the purpose of this paper: (1) How do you currently identify perinatal or postpartum depression? (2) If a mother scores high on the screening tool or is showing symptoms for depression or anxiety what are your next steps or how do you direct your patient to additional support? (3) What makes it easy to counsel patients on PPD/PPA, what makes it difficult? (4) What are the barriers in screening, and referring out for mental health concerns during this time period? Four major themes emerged regarding screening and referral practices during the perinatal period (1) Approaches to Screening and Assessment (2) Provider Responses and Interventions (3) Facilitators of Engagement and (4) Barriers at Multiple Levels. Themes, sub-themes, descriptions, and sample quotes are detailed in Table 2.

**Table 2:** Descriptions of thematic codes.

Theme and Sub-themes	Description	Sample Quotes
**Approaches to Screening and Assessment**1. Screening tools (EPDS and/or PHQ-9 2. Timing of the screening 3. Processes of assessment	There are a variety of approaches to screening and assessing perinatal mood disorders. Specifically, many providers shared the type of screening tool used (1), how often they use the screening tool or have conversations regarding mental health (2), and what the process of discussing mental health may look like in a medical setting (3).	1.“Usually the Edinburgh Postpartum Depression Scale. There have been some like PhQ-9's that I've used in the past, I would say more rarely but Edinburgh postpartum scale always for the postpartum patients” –DO 2. “We do a screening test at the beginning of pregnancy. We do it postpartum both in the hospital and when they do their follow-up visit postpartum.” –MD 3. “Typically our nurses will do the depression scale at their initial visit and then we always do them during postpartum visits and then intermittently within the midst of the pregnancy, usually, I think. Maybe like the second or third trimester, they administer again. And then during all OB visits, review their score, but then also kind of talk to them about their mood, just generally talk about postpartum depression as well.”–MD
**Provider Responses and Interventions**1. Referrals 2. Communication 3. Education 4. Treatment Options 5. Safety Planning	After identifying the mental health needs of patients or seeing a high score on a screening tool, indicating concerns for a mental health condition, providers share various methods used to respond to the needs of their patients.	1. “If they score a 10 or above, it's an automatic referral for social work.”–MD 2. “So first, I go over the results with them and kind of say this indicates that you might be struggling with some postpartum depression. How do you feel about, you know, where the scores are or what you're feeling? Do you agree with that?” –MD 3 “So, I tell women that sometimes it can be due to hormonal imbalance. After giving birth that can, you know, make the neurotransmitters and, in the brain kind of out of whack as well so it can cause a little bit of mood disorders, anxiety, depression.” –CNM 4. “I always bring up medications because I think yeah, there's pretty good safety data and like efficacy data for medications and pregnancy. So, you know, I talked to them about that risk benefits, you know, does take a while to start working” –MD 5. “So we, I usually assess for suicidal ideation or homicidal. And then if that is present. Then I send them to the ED to like get into the inpatient.” –Resident
**Facilitators of Engagement**1. Provider empathy/experience 2. Normalization 3. Willingness to share	Providers consistently shared what may make it easier to have conversations with patients about mental health concerns.	1. “I think it's easy because I had postpartum depression. So, I just quickly identify struggles, relate and share my story and be a resource to patients, so I think just personal history makes it easy for me.” –CNM 2. “I think just like including the rest of things to, you know, tell people that that's a normal thing that can happen sometimes in the postpartum or even in pregnancy.” –MD 3**. “**I do better with women who are kind of like, oh, here, let me be honest and I'm feeling really overwhelmed, when people are like straight up not in touch with how they're doing.” -MD
**Barriers at Multiple LevelsPatient** **Stigma** Disclosure Culture Motivation **Provider** **Time** Competing demands Assumptions Non-standardized processes **System** **Resources** Insurance Follow-up gaps	Providers shared what may make it more challenging to discuss mental health conditions with patients, as well as barriers to providing referrals for patients.	PATIENT 1. “**Social stigma** against mental health disorders. The fact that women see all these happy, healthy moms and that's what they want to be and so it can be hard to admit that they're struggling.”–DO 1 PROVIDER 2. “It just gets stressful, and I wonder if like some of the issues like yeah, we probably should be screening for depression at every visit or at least asking patients how their mood is. But a lot of times we **just don't even have time** with all the other stuff that needs to be done.” –MD SYSTEM 3. “**Well, we just need more resources**. Mental health is a big problem right now all over. And it's harder to get the services that you know these women need because they're so booked up. Because so many people need them.” –CNM

### Approaches to screening and assessment

Providers shared various methods to screening and assessing women in the perinatal period. Most commonly providers reported using the EPDS. Few providers reported using a PHQ-9, pending the patient. Providers reported different times to screen patients in the perinatal period. For example, one provider reported “we do the Edinburgh for any patient who is admitted into the hospital, so if they're antepartum they do the Edinburgh depression screen and then they have to do it at postpartum as well. And then when they come in for their postpartum visit, they have to do it.” One provider reported in their practice they will also do a 28 week screening, along with postpartum visits. Some providers reported they knew patients were screened postpartum but unsure if they screen during pregnancy. Additionally, the process of screening and assessing varied amongst providers. Some reported no specific protocol and informally interviewed in a clinic to assess patient mental health needs, while others reported a standardized screening process.

### Provider responses and interventions

Provider Responses and Interventions had five specific sub-themes (see Table 2). Providers shared a variety of responses to a concern for PMAD, whether that be by a high screening number or through conversation. Some providers described having a referral process in place, such as directing patients to in-house psychiatric services or immediately involving social work. Others reported having more of a conversation with the patient and furthering the screening to get a sense of overall support network for the patient. Providers noted that they often engaged in client education, which included clarifying the screening tool response and explaining the physiological role of hormones in influencing mood, behavior, and physical symptoms. Various treatment options were shared, such as medication management and counseling. When a high score on a screening tool was identified, providers prioritized immediate safety planning; assessing the presence of suicidal or homicidal ideation, clarifying intent, and determining the level of risk to ensure the safety of the individual and others.

### Facilitators of engagement

Facilitators of engagement refers to the factors that make it easier for providers to initiate and sustain conversations with patients about mental health concerns. This particular theme had four specific sub-themes (see Table 2). Overall, providers reported the (1) facilitator being provider empathy and communicating the empathy to their patients, (2) providers having a strong relationship with their patient, (3) enablers that normalizing mental health conditions may assist the conversation, (4) patients having a willingness to connect with the provider.

### Barriers at multiple levels

Barriers at multiple levels had three specific themes: patient, provider, and system barriers (See Table 2). Overall, providers reported barriers related predominantly to the patient: (1) patients do not discuss mental health needs due to the overarching stigma and comparison to other moms who may not experience, (2) disclosing is challenging for patients, however it limits assessing patient needs, (3) cultural barrier making it a challenge to discuss mental health concerns due to uncertainty of tools being validated in other languages, interpreter in the room making it difficult to understand one another, and comfortability level of patient, and (4) patient's own level of motivation, which impacts if patient ultimately seeks therapeutic services after referral. For example, **“**There are a couple of counseling groups that I will refer to, but I think it depends on how motivated the patient is to seek that care out because when you're depressed, you really don't feel like doing much”. Additionally, barriers are reported related to provider-based constraints: (1) limited time in appointments and the difficulty to dedicate it to discuss mental health, (2) competing demands in the visit making it challenging to prioritize mental health needs over physical needs, (3) assumptions made by providers that the patient is doing OK. For example, “But quite honestly, I don't know that there's that there is 1 model because everybody's susceptible and as soon as I start thinking this person is going to be okay, this one's not”. Lastly, (4) a non-standardized process through the clinic. A provider reports, “And then I think part of it's the consistency even within our own practice that I might want to do things one way and that's probably where some of the screening breaks down is oh so-and-so wants it this way, somebody else wants it this way, so we end up doing nothing because we can't get agreement on it.” Finally, several systemic barriers were noted. These include (1) lack of resources and referrals to provide to patients, (2) structural challenges, such as providers or clinics not accepting insurance and/or patients lacking insurance coverage, and (3) logistical constraints, such as childcare needs and competing demands preventing individuals from attending visits. For example, a provider reported, “…we didn't allow people to bring their babies to their postpartum visit and we saw an even larger drop in postpartum visits.”

## Discussion

In this study, we sought to explore the barriers and facilitators of identifying and addressing perinatal mood disorders from the perspective of clinicians. Our findings emphasize four key themes: (1) Approaches to Screening and Assessment (2) Provider Responses and Interventions (3) Facilitators of Engagement and (4) Barriers at Multiple Levels.

As previously discussed, PMAD's are not a rare experience and left untreated, can have detrimental consequences on the mother, infant, and entire family system. Early identification of PMAD's may result in more effective intervention and management (1, 5). Clinical practice guidelines from ACOG recommend screening for PMADs more than one time throughout pregnancy and postpartum, using a validated instrument (3). However, our findings are not consistent with ACOG guidelines. Our participants' responses to screening for PMAD's varied with some responses aligning more closely with ACOG guidelines while other participants indicated no specific method for identification of PMAD's. The EPDS is the most commonly used valid instrument among participants in this study. Relatedly, our next finding emphasized the responses and interventions of providers when a patient indicates mental health need through conversation or high scores on screening instruments. Referrals, patient education, treatment options, and safety planning were among the most commonly emphasized responses and interventions to PMAD's concerns.

When discussing mental health with clients, the providers in this study believed that having a relationship with their client helps facilitate engagement in conversations about PMAD's. This is consistent with prior literature. A 2025 study involving postpartum women found that increased knowledge about postpartum depression increased a patient's engagement in seeking help (23). Likewise, provider encouragement played a key role in promoting help-seeking behaviors among postpartum women (24). Providers indicated having a good relationship with a patient, being able to communicate with empathy, and normalizing mental health conditions all contribute to the ease of counseling patients on PMAD's. These findings are aligned with other scholars' assertions and findings that a trusting provider-patient relationship is essential for high-quality care provision (25). We also asked providers what the perceived barriers were to providing care for PMAD's. Providers reported patients experienced shame or stigma about postpartum mental health challenges. A systematic literature review examining the barriers and facilitators of help-seeking for PMAD's found that fear and stigma were barriers to seeking help for postpartum women experiencing PMAD's (26). Specifically, literature reveals new mothers are often concerned about the occurrence of potential negative outcomes because of discussing mental health concerns with women discussing a fear of their children being removed or being seen as a bad mom if they need mental health help (26). When providers are able to normalize mental health struggles in the postpartum period, they may combat some stigma and fear and reduce barriers to care. Providers and researchers have supplied various suggestions for enhanced screening, training, and resource allocation when it comes to perinatal mental health. For example, discussion around raising awareness about PMADs included ideas such as the use of waiting room pamphlets, telephone support lines, virtual medical support, as well as the creation of social media programs (11, 13). These initiatives to further educate about PMADs aim to help reduce the stigma associated with perinatal mental health challenges, which may in turn facilitate the identification and treatment of PMADs (1, 14).

System level barriers also were identified by providers, such as lack of childcare and cultural and language barriers- consistent with Jones, 2019. Additionally, our study highlights the need for providers to have extended time in patient appointments. This is consistent with prior studies indicating time as a barrier to providing mental health care in visits (27, 28). Given the complexity of PMAD's and maternal health, there is a critical need to explore mechanisms by which providers can be afforded more time per patient encounter. Finally, our providers reported concerns for lack of follow-up care. Though the 2022 World Health Organization Guidelines recommend high-quality postnatal care in the initial days after childbirth and at least three postnatal contacts within the first six weeks postpartum; follow- up was identified as a significant concern in our interviews (29). In the current study interview, providers were asked to suggest ways to improve care. Briefly, providers suggested earlier and consistent screening for PMAD's, interdisciplinary pathways, and having more visits, both in person and a virtual option. Future research should explore options for integrated and standardized care options for treatment and screening of disorders. Lastly, providers identified the limited referral options available to them; this is consistent with a meta-review in 2023 indicating resources and referrals as a barrier to women accessing perinatal mental healthcare (30). Treatment for PMAD's typically includes mental health counseling, medicine, or both (31, 32). Screening for PMAD's is a helpful start to early identification but with limited referral options, providers and patients experience constraints in treatment. Both urban and rural areas in the US are affected by the shortage of behavioral health professionals (31).

## Implications

Our findings have several important implications for both clinical practice and future research. First, our study reveals that healthcare providers have insufficient referral pathways. This is consistent with a meta-review in 2023 indicating resources and referrals as a barrier to women accessing perinatal mental healthcare (30).. Future studies should investigate strategies to optimize referral processes and resource delivery for patients during the perinatal period. A 2023 study suggests on-site assessment, as integrating mental health services in practice increases patient engagement (33). Other studies have explored the role of interdisciplinary care and its positive impact on women's health care (27, 34). The specific scoping review highlights how interprofessional education is integrated into medical training, emphasizing its positive impact on mental health within diverse health-care teams and should be considered for women's health care practice (34). Future research should explore the benefits of on-site social workers seeing patients during the perinatal period; as there are specific social workers dedicated to supporting women and families in this timeframe through the National Association of Perinatal Social Workers (NAPSW).

## Limitations

While there were many strengths to the study, limitations should be noted. This study surveyed professionals from three Midwestern states, so the views represented may not reflect those of professionals nationwide. The researchers encountered challenges in recruitment and therefore employed snowball sampling, which introduces selection bias and may restrict the generalizability of the findings. Recruitment was further limited by providers' competing professional responsibilities and time constraints, though the team made efforts to meet at their preferred time. To address these challenges, the researchers expanded recruitment efforts by contacting a variety of institutions and practice settings directly. A major limitation of this study is the homogeneity of the participant sample, which was predominantly White, female, and a reported high job satisfaction. This limited diversity may constrain the generalizability of the findings to broader populations, including individuals of different racial or ethnic backgrounds, gender identities, or those with lower job satisfaction. Future research should investigate how diverse backgrounds may influence the results. This study also depended on participants' self-reported information. Although such data can shed light on personal experiences, it may be affected by factors like memory inaccuracies, the desire to respond in socially acceptable ways, or misunderstandings.

## Conclusion

This study yields four themes providing insight into how providers screen, respond to, and engage patients around PMAD's, while also identifying multilevel barriers that limit care. These themes are: (1) Approaches to Screening and Assessment, (2) Provider Responses and Interventions, (3) Facilitators of Engagement and, (4) Barriers at Multiple Levels. Despite these contributions, continued research is needed with more providers throughout the US. Additionally, incorporating patient voices is critical to use as a tool to ensure provider reports align with patient experiences. To advance the state of perinatal mental health, we must seek to reduce the barriers and increase facilitators.

## Data Availability

The raw data supporting the conclusions of this article will be made available by the authors, without undue reservation.
